# Lifestyle and Selected Issues Related to Sexual Health: The Importance of Specialist Care in Balneology, Dietetics, and Physiotherapy

**DOI:** 10.3390/jcm15010307

**Published:** 2025-12-31

**Authors:** Agata Puszcz, Paulina Kozłowska, Justyna Wójcik, Anna Morawska, Małgorzata Wójcik, Katarzyna Plagens-Rotman, Monika Englert-Golon, Jakub Mroczyk, Małgorzata Mizgier, Ewa Jakubek, Magdalena Pisarska-Krawczyk, Stefan Sajdak, Klaudyna Madziar, Witold Kędzia, Grażyna Jarząbek-Bielecka

**Affiliations:** 1 Sexology and Clinical Psychology Student Research Group, Poznań University of Medical Sciences, 70 Bukowska Street, 60-812 Poznań, Poland; 86056@student.ump.edu.pl (P.K.); 83469@student.ump.edu.pl (J.W.); 85772@student.ump.edu.pl (A.M.); 2 Department of Physiotherapy, Faculty of Physical Culture in Gorzow Wielkopolski, Poznań University of Physical Education, 13 Ewaryst Estkowski Street, 66-400 Gorzów Wielkopolski, Poland; malgo_wojcik@interia.pl; 3 Clinic of Gynecology, Poznań University of Medical Sciences, 33 Polna Street, 60-535 Poznań, Poland; kpr.pielegniarstwopolskie@interia.eu (K.P.-R.); jakub.mroczyk@gmail.com (J.M.); ssajdak@ump.edu.pl (S.S.); klaudyna.madziar@gmail.com (K.M.); witold.kedzia@ump.edu.pl (W.K.); grajarz@tlen.pl (G.J.-B.); 4 Division of Gynaecological Oncology, Department of Gynaecology, Poznan University of Medical Sciences, Polna 33, 60-535 Poznań, Poland; mgolon@ump.edu.pl; 5 Department of Sports Dietetics, Faculty of Health Sciences, Poznań University of Physical Education, 27/39 Queen Jadwiga Street, 61-871 Poznań, Poland; mizgier@awf.poznan.pl; 6 Faculty of Health Sciences, Poznań University of Medical Sciences, 70 Bukowska Street, 60-812 Poznań, Poland; ejakubek.x@gmail.com; 7 Department of Social Medicine and Public Health, Faculty of Medicine and Health Sciences, Calisia University, 62-800 Kalisz, Poland; 8 Nursing Department, Calisia University, 62-800 Kalisz, Poland; magmp@op.pl

**Keywords:** sexual dysfunction, women’s health, balneotherapy, physical therapy modalities

## Abstract

**Background/Objectives**: Sexual health is shaped by lifestyle factors alongside biomedical determinants. This review synthesises evidence on physiotherapy, balneology/peloidotherapy, and diet therapy as preventive and therapeutic adjuncts for female sexual dysfunctions and related gynaecological conditions. **Methods**: A structured narrative review of PubMed and Google Scholar (June–July 2025) was conducted by three independent reviewers using predefined keywords in English and Polish. Case reports, preprints, and studies before 2015 were excluded. From 7322 records, 47 studies met the inclusion criteria for qualitative synthesis. **Results**: Physiotherapy—particularly pelvic floor muscle training, multimodal manual therapy, neuromuscular electrical stimulation (including PTNS), magnetostimulation, short-wave diathermy, and capacitive–resistive monopolar radiofrequency—was consistently associated with reductions in dyspareunia, chronic pelvic pain, and urinary symptoms, with parallel improvements in sexual function and quality of life. Balneological procedures (brine baths/irrigations, crenotherapy, selected radon/sulphide/iodine–bromine applications) and peloidotherapy demonstrated analgesic, anti-inflammatory, and perfusion-enhancing effects, with signals of benefit in vulvodynia, endometriosis, and infertility support. Dietary measures—higher fruit intake (notably citrus), adequate vitamin D, targeted omega-3 use in PCOS, a Mediterranean dietary pattern, and prudent red-meat limitation—were associated with favourable endocrine–metabolic profiles and, in selected contexts, reduced disease risk. **Conclusions**: Integrating lifestyle–medicine modalities with standard care may meaningfully prevent and manage female sexual dysfunctions by addressing pain, perfusion, neuromuscular control, and endocrine–metabolic drivers. Implementation frameworks and high-quality trials are warranted to refine indications, dosing, and long-term effectiveness.

## 1. Introduction

According to the World Health Organization, sexual health constitutes a complex integration of biological, emotional, cognitive, and social factors that play a significant role in shaping personality, interpersonal relationships, and the capacity to form emotional bonds and love [1]. This concept fits within the broader context of health promotion, encompassing efforts to popularize a health-promoting lifestyle, balanced nutrition, and physical activity. Within a holistic model of treating sexual dysfunctions, it is appropriate to consider diverse therapeutic strategies that include not only pharmacotherapy and psychological interventions. Sexual health is determined not only by general physical and psychological condition but also by several lifestyle-related factors. These include physical activity, diet, daily routine, and the quality of the partner relationship. Given this multidimensional background, integrating core therapy with elements of lifestyle medicine appears justified, including natural methods, an appropriately tailored diet, and physical exercises. A similar approach should be applied to the treatment of infertility disorders, which often result from sexual dysfunctions, co-occur with them, or conversely, constitute their primary cause.

The use of balneotherapy, physiotherapeutic interventions, and a properly balanced diet may significantly improve the sexual quality of life in women. These effects may be direct, occurring through increased libido, greater sexual satisfaction, improvement in potency, and alleviation of pain during intercourse (dyspareunia). They may also be indirect, arising from the mitigation of symptoms of chronic diseases that contribute to reduced sexual satisfaction. Particular attention should be paid to conditions such as chronic pain in the lesser pelvis and the lumbar spine, pelvic organ prolapse, endometriosis, vulvodynia, and vaginismus, which are significant factors limiting sexual activity.

It is also warranted to consider the potential of the aforementioned therapeutic interventions in the context of improving fertility and supporting the sexual health of postmenopausal women.

The present review provides an analysis of the potential utility of physiotherapeutic, balneological, and dietary methods in the treatment of sexual disorders. It takes into account both their direct therapeutic applications and the indirect ways in which they affect sexual health. These involve pain reduction, mitigation of local inflammation, and control of underlying conditions such as vulvodynia or endometriosis, as well as the improvement of parameters related to fertility.

The primary objective of this review is to demonstrate the importance of physiotherapy, balneotherapy, and diet therapy in the context of sexual health as a complement to classical therapeutic methods.

## 2. Materials and Methods

Three independent reviewers conducted searches of the medical databases PubMed and Google Scholar, using the following keywords and MeSH terms: gynecology or sexology, appearing in the article together with the terms: fizjoterapia (=physiotherapy, 1142 results), fizykoterapia (=physical therapy, 1956 results), peloidoterapia (=peloid therapy, 3 results), balneologia (=balneology, 27 results), dietoterapia (=dietotherapy, 2450 results), dieta (=diet, 1744 results) (in both English and Polish). The exclusion criteria were the following: case reports, works prior to the peer-review process (preprints), and works older than ten years (before 2015). While the inclusion of additional databases such as Scopus or Web of Science could have further expanded the search, PubMed and Google Scholar were deemed sufficient to capture the core literature relevant to the scope of this narrative review.

In this article, the authors analyse aspects of the use of physiotherapy, diet, and balneoclimatology in sexology. The literature review was conducted from June to July 2025. The collected items were then assessed for compliance with the established inclusion and exclusion criteria based on abstract analysis.

**Figure 1:**
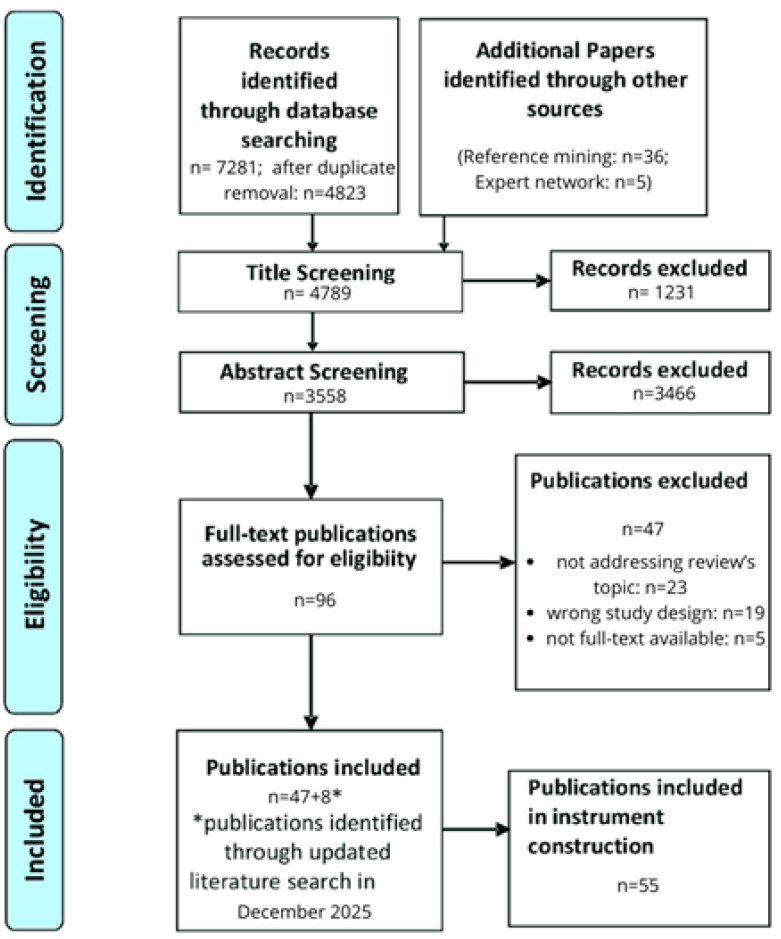
A PRISMA flow diagram of the literature selection process.

Of the 7322 articles initially qualified for further analysis, 2458 were excluded due to duplication, and 1231 due to failure to meet the aforementioned criteria. The remaining articles underwent abstract analysis, of which 96 publications were qualified for further, detailed analysis. All included articles were evaluated in terms of form, method, type of therapy applied, therapeutic objective, and conclusions resulting from the conducted studies. Ultimately, 55 articles were included in the review (Figure 1).

## 3. Results and Discussion

Health, including sexual health, is also a reflection of an individual’s lifestyle. Healthy sleep, a healthy, balanced diet rich in vitamins and minerals, regular physical activity, and the avoidance of stimulants have a positive effect, for example, on libido and the overall condition of the body [2].

According to the World Health Organization, sexual health is the integration of the biological, emotional, intellectual, and social aspects of sexual life, which are important for the positive development of personality, communication, and love [1].

Diet may be crucial for libido; indeed, it is even claimed: ‘Healthy eating therefore also means successful sex’ [3].

The goal of the prevention of many diseases is to ensure good quality of life, including functioning in the sexual sphere. Sexual health has a strong impact on overall well-being. However, during the medical interview this aspect is often omitted by patients, and there is a need for physicians—as well as dietitians, physiotherapists and psychologists—to enquire about these issues. Sexual dysfunctions, broadly speaking, are not an uncommon problem and concern both men and women [4]. Assuming that a satisfying intimate life is one of the foundations of successful partnerships, this becomes a problem in a broad sense, as sexual health problems markedly reduce quality of life and disrupt even everyday functioning. It turns out that a deterioration in sexual health is influenced by being overweight, obesity, diabetes, and cardiovascular diseases. Other contributing factors include the increasingly frequent occurrence of metabolic syndrome, chronic kidney disease, and neoplastic diseases [5,6,7,8,9]. Some of these conditions may arise as a result of an unhealthy lifestyle and an improper diet, which also results in a reduction in sexual attractiveness.

In addition, an unhealthy lifestyle, including tobacco smoking, has a highly adverse effect on sexual attractiveness and sexual function [10]. Impairment of sexual function—for example, issues related to achieving and maintaining an erection—also results from cardiovascular diseases, such as ischaemic heart disease or arterial hypertension [7].

It is worth noting that many medical problems that may negatively affect sexual health are diet-related. One of the main causes of their occurrence is an inappropriate diet, and diet-related diseases may co-occur, which is even more dangerous. In the course of these diseases, narrowing or even damage to blood vessels may occur, which compromises blood transport to and from the genital organs. This, in turn, contributes to erectile problems in men, because an insufficient volume of blood reaching the penis may impede or even preclude an erection [11]. It is worth emphasizing that successful sexual intercourse is possible due to the coordinated action of psychological factors and essential physiological functions, including the endocrine, vascular, nervous, and muscular systems. When lifestyle is characterised by an unhealthy diet, stress and lack of physical activity, it may substantially disrupt central nervous system functioning, consequently impairing the production of hormones and neurotransmitters involved in sexual performance [12,13]. It is important to draw attention to the fact that, as a consequence of an improper lifestyle, obesity readily develops, and it is often accompanied by disorders of lipid and carbohydrate metabolism, as well as arterial hypertension and endocrinopathies. These diseases may result in problems related to functioning in the sexual sphere, together with a loss of a sense of sexual attractiveness and reduced self-esteem in both women and men [12,13].

Secondarily, there may even arise a fear of criticism of the obese person’s appearance, including by a partner, which leads to reluctance to engage in sexual activity and disrupts the quality of partner relationships [14].

It is therefore advisable, in preventive activities—both in the context of general health and sexual health and of proper development—to promote the ‘Healthy Eating and Physical Activity Pyramid’. In the context of therapy for sexual dysfunctions, a holistic treatment model is important, encompassing a range of therapeutic strategies that include not only pharmacotherapy and psychological interventions, but also physiotherapy, diet therapy, and balneotherapy.

### 3.1. The Significance of Balneotherapy and Peloid Therapy

Balneotherapy encompasses a wide range of therapeutic procedures employing natural medicinal resources, such as mineral waters (e.g., brine baths, vaginal irrigations, crenotherapy), therapeutic gases (including ozone), and peloid.

The literature indicates that balneotherapy confers benefits in alleviating climacteric symptoms, in chronic inflammations (of the urinary tract, the lesser pelvis, the vulva, and the vagina), in the treatment of vulvodynia and endometriosis, and potentially also infertility. Moreover, a direct, positive effect on sexual health has been demonstrated through the reduction of pain during intercourse [15].

Brines exhibit antiseptic and immunostimulatory effects. In brine baths, solutions containing sodium chloride (most commonly) and salts of calcium, potassium, magnesium, or other elements are used, with the temperature of the solution kept between 33 and 40 °C. In patients immersed in a brine bath, an increase in blood electrolyte concentrations is observed, which in turn leads to activation of cutaneous receptors, interoceptive and autonomic responses, and stimulation of the endocrine glands. The secretion of histamine, adrenaline, and serotonin increases. The effects of these phenomena include vasodilatation, improved skin perfusion, muscle relaxation, reduced excitability of sensory and motor nerves, and enhancement of non-specific immune defence [15,16].

Brine irrigations are performed on a gynaecological chair using specialised cannulas with a tip enabling controlled infusion of the solution and its even distribution within the vagina. Crenotherapy is the oral administration of brine waters [16]. Brine therapies have been applied primarily in the treatment of chronic pain syndromes. These include chronic pelvic pain (CPP), pelvic pain of orthopaedic aetiology, and vulvodynia. It should be noted that CPP is a well-established risk factor for sexual dysfunctions, as it involves dyspareunia and disorders of vaginal perfusion and lubrication. Such symptoms can lead to difficulties in achieving orgasm (anorgasmia) and arousal disorders (hypolibidaemia) owing to anticipatory fear of pain [16]. Vulvodynia may give rise to burning, stinging, tingling, or a sensation of wounds within the vulva, even with minimal touch, and may even result in secondary vaginismus. The effectiveness of brine baths has also been demonstrated in cases of primary dyspareunia not associated with a chronic inflammatory process [15].

Selected forms of balneological therapy—such as brine baths and irrigations, as well as crenotherapy—are used in the treatment of endometriosis [16]. It is worth emphasising that these are the only spa procedures recommended in this group of patients, since peloid-based treatments—owing to the presence of phytoestrogens—may potentially exacerbate symptoms and adversely affect the course of the disease. During menstruation, endometriotic foci undergo ischaemic transformations, which lead to chronic inflammation, adhesion formation, and intensification of pain, especially during sexual intercourse. However, the available scientific evidence remains limited. Further well-designed studies with larger cohorts are necessary to identify the most effective approaches in the management of endometriosis. Prolonged hormonal therapy with gonadotropin-releasing hormone (GnRH) analogues and combined oral contraceptives often results in disturbances of libido. Women with endometriosis also frequently experience chronic pain and psychological stress. These factors can lead to depression, anxiety, and reduced self-esteem, which in turn leads to a deterioration in sexual quality of life and reduced satisfaction with intimate relationships [16].

Among other balneological procedures used in the treatment of gynaecological conditions, a special place is occupied by radon baths at concentrations in the range of 40–200 nCi/L [15]. The beneficial effect of radon in the therapy of chronic inflammatory conditions is attributed to radiation hormesis, which posits that low doses of alpha radiation stimulate the body’s defence responses and support tissue repair processes [17]. Radon baths are employed in the treatment of endometriosis [15].

Other brine baths that have found application in the treatment of chronic gynaecological diseases that diminish the quality of sexual life include the following: sulphide baths (sulphide concentration 100–150 mg/L, recommended for the treatment of vulvodynia), iodine–bromine baths (for chronic inflammations), and arsenic baths [15].

The research team of Dias et al. (2023) draws attention to the potential of ozone saunas in the treatment of infertility [18]. The medical experiment aimed to test whether combining ozone sauna therapy (OST) and pulsed electric field therapy (PEMF) could improve infertility treatment outcomes in women with diminished ovarian reserve, both in vivo and in vitro in granulosa cells (GC). In the in vitro trial, OST with PEMF increased aromatase expression fivefold. In the in vivo trial, the number of embryos formed was significantly higher following OST+PEMF. Additionally, an improvement in endometrial thickness (EMT) was observed, which is an important indicator of pregnancy success. The study has several limitations that might reduce the strength of the conclusions. Although the significant improvement in embryo number and increase in EMT were reported, the outcomes such as pregnancy and implantation rate were not assessed—most participants are continuing embryo banking due to the severity of their diminished ovarian reserve. In addition, most embryos were frozen at the cleavage stage, only four genes in GCs were assessed, and only mRNA expression was measured. Peloid, i.e., medicinal peat, plays a significant role in the management of gynaecological disorders. It is particularly useful in chronic inflammatory diseases of the genital organs, in alleviating climacteric symptoms, and as an adjunct therapy in infertility treatment [18].

Treatment with peloid is referred to as peloidotherapy. Peloidotherapy procedures are carried out in various forms, depending on clinical need. The most common are wraps, sitz baths, peloid tampons, peloid ‘briefs’, and rectal instillations [19]. Medicinal peloid contains a variety of bioactive compounds, including humic substances, sulphides, and other organic components. The latter in particular exhibit properties similar to the action of oestrogenic hormones. Appropriate preparation of peloid yields a plastic, dense mass characterised by high heat capacity and excellent sorptive properties. Following absorption through the skin and mucous membranes, the active compounds of natural peloid show a broad range of therapeutic effects. These cover anti-inflammatory, desensitising, resorptive, anti-oedematous, and bactericidal actions, together with the inhibition of exudative and infiltrative processes. They further improve blood rheology and tissue perfusion and help regulate the hypothalamic–pituitary axis and ovarian endocrine function [15,19,20]. As demonstrated in studies, peloid may influence the concentrations of sex hormones such as oestradiol, oestriol, progesterone, and testosterone, and may also contribute to a reduction in blood insulin levels. These properties make peloidotherapy applicable as an adjunct method in the treatment of infertility, especially in cases of endocrinological aetiology [21].

The physicochemical processes occurring during peloid baths and packs are based on ion-exchange properties and on the ability of humic acids to penetrate the skin, which enables detoxification of the organism. At the same time, it has been observed that peloid exerts a beneficial effect on the functioning of the autonomic nervous system. During the procedure, sympathetic tone increases, whereas after its completion there is prolonged stimulation of the parasympathetic branch, which may consequently bring relief from symptoms characteristic of the menopausal period [22]. Peloid also exhibits a stimulating effect on ovarian function. This may occur both locally—via thermal heating of the gonads—and systemically, through the penetration of oestrogen-like bioactive compounds and their action on the hypothalamic–pituitary axis [19].

Vaginal peloid tampon therapy plays an important role in treating chronic and recurrent inflammations of the adnexa and the vagina. This therapy involves the intravaginal application of peloid in the form of a tampon. For these procedures, a specially prepared preparation—peloidin—is used, obtained from peloid milled for several hours to achieve particles with a diameter below 0.01 mm. This material is then heated to body temperature or slightly higher (up to 44 °C) and exhibits high permeability, as its active components readily penetrate the mucous membrane [15]. The degree of comminution of the peloid mass affects the efficiency of substance exchange between it and the patient’s tissues—the finer the fraction, the more effective the diffusion. These procedures last from ten to twenty minutes and are performed three to five times per week. The tampon is inserted on a gynaecological chair, and after the procedure it is removed by thoroughly rinsing with sterile water at a temperature of 38–40 °C [19]. Habek et al. (2020) draw attention to the potential of peloid tampons in the treatment of lichen sclerosus et atrophicus of the vulva [15]. It is worth noting that lichen lesions lead to narrowing of the vaginal introitus and the formation of adhesions, which are a cause of dyspareunia [15].

The team of Min et al. (2020) conducted an experiment in Korea in a group of patients (n = 16) with CPP [23]. The balneotherapy programme comprised two brine baths and two peloid packs during a 5-day course. A reduction in pain symptoms was observed in the patients, as well as decreases in the inflammatory markers interleukin-1 (IL-1) and tumour necrosis factor alpha (TNF-α) in serum. It should be noted that the study has a small sample size and did not include a control group. In addition, the authors highlight the difficulty of completely eliminating the placebo effect in research on balneotherapy [23].

Peloid packs are a recognised form of treatment for CPP, as well as chronic inflammation of the adnexa. The procedure involves applying a peloid pulp heated to 45 °C to the lower abdomen or sacral area. The application thus prepared is wrapped in a sheet, foil, and a blanket in order to maintain the temperature. A session lasts from 20 to 30 min, with the thermal effect developing gradually and persisting for several hours, ensuring vasodilatation in the deeper tissue layers. Packs made from volcanic peloids (fango) also have potential in the treatment of infertility and sexual disorders such as dyspareunia and hypolibidaemia [15,19].

In physiotherapy practice, peloid at iontophoresis is also used. The procedure consists of applying high-grade peloid to the sacral region in a layer approximately 3 cm thick, followed by the application of a cathode conducting a galvanic current of 10–20 mA. The session lasts from fifteen to twenty minutes and should be repeated three to four times per week [24].

### 3.2. The Significance of Physiotherapy

Disorders of pelvic organ support and the associated urogynaecological problems constitute a form of disability that limits physical and sexual activity. Data indicate that over 50% of women with these disorders exhibit hypolibidaemia or alibidaemia and feel less attractive and avoid sexual contact, which disrupts partner relationships. This state is influenced by discomfort during intercourse resulting from hypo-estrogenic atrophic changes, irritation of the genital organs by urine (climacturia), and recurrent inflammations or infections of the urogenital tract. It is therefore worth seeking effective dietary and physiotherapeutic interventions as adjunctive treatment. In the management of urogynaecological disorders, a particular role may be played by manual therapy, including pelvic floor exercises, as well as physical medicine procedures such as electrostimulation, magnetostimulation, monopolar radiofrequency, and shortwave diathermy [19,24].

Pelvic floor muscle exercises are the basic physiotherapeutic method in urogynaecology, used prophylactically and therapeutically for disorders of pelvic organ support, urinary incontinence, and pain syndromes such as dyspareunia or vulvodynia. They constitute first-line treatment in women with various forms of urinary incontinence.

In a study by Weber-Rajek et al. (2020), patients with stress urinary incontinence were instructed for 12 weeks to contract the pelvic floor muscles using a technique involving activation of the transversus abdominis muscle [25]. This therapy led to an alleviation of symptoms, resulting in improved quality of life.

In another study, a programme of alternating long-hold and rapid pelvic floor contractions performed in various positions produced similar effects. It also favourably influenced sexual function, as assessed using the Female Sexual Function Index (FSFI) [26]. The cited well-designed studies show that different pelvic floor muscle training techniques, although distinct, can effectively alleviate the symptoms of urinary incontinence. Taken together, the findings indicate robust evidence that PFMT (Pelvic Floor Muscle Training) effectively alleviate symptoms of stress urinary incontinence [26].

Pelvic floor muscle exercises are also effective in the therapy of pain and sexual dysfunctions. In perimenopausal women with dyspareunia, a training programme encompassing therapy of fascial trigger points in the pelvic floor muscles, the abdominal diaphragm, the piriformis, and the iliopsoas in five one-hour sessions. This intervention resulted in pain reduction, improved muscle function, and enhanced sexual function [27]. However, the study had several limitations, including the absence of participant blinding, a small sample size, and a homogenous study population. Given that this study constitutes a valuable addition to the field, further high-quality research is warranted [27].

Similar effects are achieved in patients with endometriosis and chronic pelvic pain with vulvodynia—regular exercises relax the pelvic floor muscles and reduce pain symptoms [28]. The study is strengthened by its randomised design, blinded outcome assessment, and use of a standardized physiotherapy protocol. Nevertheless, the small sample size and lack of participant blinding limit the overall robustness of the evidence [28]. In provoked vestibulodynia (a form of vulvodynia), a combination of several techniques proved effective, including pelvic floor contractions, stretching exercises using a dilator, fascial therapy, and neuromuscular re-education. A 10-week course of therapy yielded better outcomes than the topical use of lignocaine. It led to pain reduction, improved sexual function, and decreased intercourse-related stress [29]. Evidence from this study can be considered robust, due to the use of a randomised design and long-term follow-up, as well as high participants’ adherence to the procedures. Although participant blinding was not feasible because of the nature of the interventions, potential bias was reduced through the use of blinded assessors. Taken together, these methodological features strengthen the credibility and overall rigor of the evidence generated by this trial. Biofeedback can contribute meaningfully to the therapeutic process by facilitating control and assessment of correct exercise performance. As a result, women can better modulate muscle activity and improve the overall effectiveness of training [30]. A key limitation of the study was the absence of objective outcome measures, including EMG and perineometer evaluations of pelvic floor muscle strength and endurance [30].

Both individual training under the supervision of a physiotherapist and group classes or exercises performed independently at home yield positive results, making this method readily accessible and applicable for most women. However, it is important that the training programme be prepared and tailored by a specialist, who will also assess whether the patient has any contraindications to exercise. This ensures that the therapy is safe and effective [31].

Electrostimulation, encompassing techniques such as neuromuscular electrical stimulation (NMES) and transcutaneous electrical nerve stimulation (TENS), is used in gynaecology both for pain relief and for stimulation of the pelvic floor muscles. Its analgesic effect is explained by the gate control theory, according to which stimulation of large-diameter nerve fibres inhibits the transmission of pain stimuli by small fibres. Regular electrostimulation of the pelvic floor muscles, in turn, induces their remodelling by increasing the proportion of slow-twitch fibres, which improves tone and contraction strength, as well as the stabilisation of organs [21]. Vaginal probes, rectal probes, and surface electrodes are used in therapy [32]. Effectiveness depends on proper diagnosis of the patient and, depending on the condition, selection of appropriate stimulation parameters, especially pulse frequency. Electrostimulation helps in the treatment of voiding disorders such as urge urinary incontinence, overactive bladder and neurogenic conditions, supporting the sphincter and detrusor muscles and thereby improving control of micturition [32].

Particularly good results are achieved with percutaneous tibial nerve stimulation (PTNS), which—as shown in studies by Musco et al. (2016)—improves not only urinary symptoms but also sexual function, even in patients who had not previously reported difficulties in this sphere [33]. However, the findings should be interpreted with caution due to the lack of randomized controlled design. Electrostimulation is also used in the treatment of chronic pelvic pain and dyspareunia in women with endometriosis, bringing pain reduction and improved quality of life, including sexual quality of life [15,16]. Additionally, TENS may effectively support hormonal therapy in pelvic pain syndrome associated with deeply infiltrating endometriosis [34]. Although positive effects were reported, the study is limited by the short follow-up period, which is insufficient given the chronic nature of the condition [34].

Magnetostimulation is another physiotherapeutic method used in urogynaecology that employs a variable magnetic field to stimulate the pelvic floor muscles. Its action involves activation of the sodium–potassium pump and regulation of depolarisation of motor neurones, which elicits muscle contractions in the innervated area. Within the pelvis, this effect is focused mainly on the motor fibres of the pudendal and visceral nerves. For therapeutic purposes, various types of devices emitting a magnetic field are used. One of the simpler forms comprises small applicators that can be placed in underwear in the perineal region. These allow direct exposure of the pelvic organs to the magnetic field, which may be continuous or periodic depending on the indication [21]. A more advanced form of therapy is extracorporeal magnetic innervation (ExMI) delivered via a so-called magnetic chair. This device emits a magnetic field with a high flux density of up to 2 tesla and an adjustable frequency from 10 to 50 Hz [25]. ExMI focuses on the stimulation of the sacral nerves, which control the pelvic floor muscles and the functions of the bladder, urethra, and rectum, thereby supporting their proper functioning. The therapy is painless and convenient, as it does not require undressing or the use of vaginal or rectal probes [19,32]. Magnetostimulation also helps reduce pain in the lumbosacral spine, especially when combined with laser therapy, which improves microcirculation and increases myelination of nerve fibres and the enzymatic activity of tissues [35]. The study provides preliminary evidence based on validated pain and disability measures; however, the small sample size, non-randomized and unblinded design, and short-term follow-up limit the robustness of the findings [35].

Shortwave diathermy is a procedure that uses an electromagnetic wave at a frequency of 27.12 MHz to produce deep tissue heating. The resulting rise in temperature accelerates biochemical reactions, stimulates metabolism, and provides analgesic and anti-inflammatory effects. Its advantage over superficial heat sources is its ability to warm deeper organs [21]. It is used, among other indications, for chronic adnexitis and pelvic pain, including in the course of chlamydiosis or gonorrhoea [36]. The study’s results are comparable to findings reported in other studies; however, its non-randomized and unblinded design, lack of a sham control, and short-term follow-up limit the strength of the evidence [21].

Capacitive–resistive monopolar radiofrequency (CRMRF), similar to shortwave diathermy, acts by generating heat in deep tissues, improving perfusion and metabolism and supporting regenerative processes. It differs, however, in the use of a lower electromagnetic field frequency (448 Hz), which allows a thermal effect to be achieved without the risk of excessive overheating, and therefore does not require cooling systems. Evidence for the effectiveness of CRMRF in the treatment of chronic pelvic pain is provided by the study of Carralero-Martínez et al. (2022), in which a series of ten treatments significantly reduced pain in patients with chronic pelvic pain syndrome [37]. While the study provides preliminary evidence of effectiveness, its small sample size and short-term follow-up limit the strength of the findings; nevertheless, its randomized design and use of validated outcome measures support the reliability of the reported results [37]. The techniques cited may be combined in a multimodal approach, which translates into greater effectiveness in treating disorders that affect women’s sexual health, such as dyspareunia. Integrated use of manual methods, physical therapy, and pelvic floor muscle exercises promotes symptom reduction, improvements in sexual function, and overall quality of life [30].

### 3.3. The Significance of Proper Diet

Dietary habits may constitute both a protective factor and a factor increasing the risk of developing disorders that affect sexual health. The literature lists numerous links between the intake of specific nutrients and the development of diseases of the reproductive system, such as uterine fibroids, endometriosis, and polycystic ovary syndrome (PCOS). In the treatment of these conditions, an important recommendation is a change in lifestyle, including dietary modification.

The most frequently analysed components of the diet include vegetables and fruit. The team of Shen et al. (2016) observed reduced consumption of broccoli, cabbage, Chinese cabbage, tomatoes, and apples among women diagnosed with uterine myomas [38]. This study provides valuable insights into risk factors for uterine myomas due to its large sample size and clear case definition, but selection bias, recall bias, potential confounding, and limited generalizability limit the strength of the evidence [38]. Uterine myomas are common benign neoplastic lesions in the female population. Depending on the size of the tumour, they may cause lower abdominal pain, constipation, and difficulty in urination, as well as dyspareunia and infertility.

The consumption of vegetables and fruit may also have a protective effect with regard to endometriosis. Chronic pelvic pain, painful menstruation, and dyspareunia associated with the disease significantly reduce patients’ physical and psychological well-being, as well as their sexual satisfaction. A diet rich in fruit is associated with a lower incidence of laparoscopically confirmed endometriotic foci. This relationship is particularly evident in the case of citrus fruit—there was a 22% reduction in the risk of endometriosis in women consuming one portion of citrus fruit per day. In the case of vegetables, a similar correlation was not found. However, daily intake of cruciferous vegetables may be associated with a higher risk of endometriosis compared with consumption less frequent than once a week [39]. This prospective cohort study provides robust observational evidence with a large sample size, repeated validated dietary assessments, and adjustment for multiple confounders; however, residual confounding, self-reported outcomes, and limited generalizability may restrict causal interpretation [39].

Furthermore, the team of Schwartz et al. (2022) indicates a higher risk of endometriosis among women following a diet with a high intake of fibre derived from vegetables [40]. Strengths of the study include validated repeated dietary assessments, a prospective design, and well-validated laparoscopic diagnoses, while limitations involve self-reported dietary intake and the potential presence of a few undiagnosed endometriosis cases in the comparison group [40].

Among vitamins, the greatest protective effect against the occurrence of uterine fibroids has been demonstrated for vitamin D, particularly among white women [41]. The team of Ciavattini et al. (2016) conducted a study involving 208 women with vitamin D deficiency who had been diagnosed with uterine myomas [42]. In 53 individuals with a small fibroid burden, adequate vitamin D supplementation was implemented. Twelve months after the initial diagnosis, a lower proportion of disease progression, to an extent requiring surgical intervention or pharmacological treatment, was noted. In patients who properly supplemented with vitamin D, no change in the dimensions and number of myomas was noted. In contrast, women who did not receive supplementation experienced a slight increase in lesion size, necessitating appropriate treatment [42]. As noted by the authors, the study should be considered preliminary due to its non-randomized design, small sample size, and reliance on patient compliance. Further well-designed, randomized studies are warranted. Nevertheless, the study’s use of standardized measurements and year-long follow-up represents a methodological strength [42].

In a study conducted in a population of women of Chinese nationality, the mean serum concentration of 25-hydroxyvitamin D (25OHD) in participants with uterine fibroids was compared with that in women without such a diagnosis. The prevalence of fibroids in women with 25OHD deficiency was significantly higher than in women with a normal 25OHD concentration (>20 ng/mL) [43]. However, the study’s observational design and its conduct at a single centre limit the strength of the evidence [43].

Similar observations were made when examining 25OHD concentrations in women with endometriosis. The 25OHD concentration was significantly lower in serum samples from patients with a severe form of the disease compared with healthy women or those with a mild form of endometriosis [44]. The study’s strengths lie in its combination of in vitro experiments and controlled serum measurements with multiple molecular endpoints, while its limitations, including the use of ESCs from endometriomas only and potential viability effects on secretions, limit causal inference and generalizability [44].

High-dose vitamin D supplementation has also shown a beneficial effect in women with PCOS accompanied by insulin resistance. In a study, patients taking 4000 IU of vitamin D together with metformin daily for 12 weeks achieved favourable outcomes. These included reductions in total testosterone, insulin, fasting glucose, and high-sensitivity C-reactive protein (hs-CRP). Additionally, a decrease in the free androgen index (FAI) and a reduction in the severity of hirsutism were observed. The study group also exhibited an increase in the mean concentration of sex hormone-binding globulin (SHBG) and total antioxidant capacity (TAC) compared with the group taking lower doses of vitamin D (1000 IU daily) and the placebo group [45]. Despite several limitations, including a small sample size, short follow-up, single-centre setting, and concurrent metformin use, the study’s randomized, double-blind, placebo-controlled design, dose comparison, and objective biochemical measurements enhance its robustness [45].

In a 5-year prospective study, Brasky et al. (2020) assessed the relationship between dietary fat intake and the incidence of uterine fibroids [46]. Total fat consumption and individual fractions (saturated, monounsaturated, polyunsaturated, and trans) showed no significant correlation with the proportion of women diagnosed with fibroids. Intake of omega-3 fatty acids, however, was associated with a higher incidence of uterine fibroids—the dietary share of docosahexaenoic acid (DHA) correlated with a 49% higher incidence. However, possible inaccuracies in dietary reporting and a focus on a specific population limit causal interpretation and generalizability [46].

The study by Di Nicuolo et al. (2021) provides evidence that alpha-lipoic acid (ALA) reduces the migration and invasion of endometriosis cell lines, which may lead to inhibition of disease progression [47].

Studies indicate that a diet rich in omega-3 fatty acids may exert a beneficial effect in patients with PCOS. It appears to reduce concentrations of CRP, malondialdehyde (MDA), luteinising hormone (LH), and total testosterone (TT). It may also increase TAC and the concentration of SHBG [48].

A favourable element of diet therapy for women with endometriosis is limiting the consumption of red meat. Research data confirm the adverse effect of its intake on the risk of developing endometriosis, particularly in the population of women who have never reported infertility [49]. The study’s large prospective cohort design with laparoscopically confirmed endometriosis strengthens the evidence; however, dietary measurement errors remain a potential limitation [49]. Avoiding red meat in the diet is also associated with a lower risk of erectile dysfunction in men [3].

To date, no unequivocal effect of milk and dairy products on the risk and growth of uterine fibroids has been demonstrated. In a prospective cohort study conducted by Gao et al. (2018), frequent intake of cow’s milk and soya in the diet was found to be a potential factor in the development of uterine fibroids [50]. Owing to conflicting results obtained in earlier studies, further analyses are needed to determine the role of these products in the aetiopathogenesis of uterine fibroids [51]. Consumption of dairy products during adolescence may, however, reduce the risk of developing endometriosis at a later age [52]. The findings are derived from a large prospective cohort with laparoscopically confirmed endometriosis, yet the evidence is tempered by potential recall bias and residual confounding [52].

Excess body mass, constituting an element of the metabolic syndrome and often co-occurring with PCOS, may cause disturbances in body image leading to anxiety and avoidance of sexual activity, irrespective of patients’ hormonal profile [53]. The Mediterranean diet may be an effective tool in the weight-loss process. Owing to its anti-inflammatory, antioxidant, and vasodilatory properties, this dietary model may also improve sexual function in both women and men. Moreover, studies indicate a beneficial effect of the Mediterranean diet on sperm motility [54]. However, the causal interpretation is limited due to the cross-sectional design, potential residual cofounding, self-reported diet, and focus on healthy men. By contrast, the study’s objective semen analysis and validated dietary assessment strengthen the evidence.

In summary, healthier lifestyle patterns are associated with higher sexual satisfaction, which may contribute to overall well-being [55].

## 4. Conclusions

Effective support for women’s sexual health requires the integration of classical treatment methods with elements of lifestyle medicine. The inclusion of physiotherapy, balneotherapy, and diet therapy may significantly improve sexual quality of life by reducing pain, enhancing perfusion and muscle function, and optimising metabolic and hormonal parameters.

The use of brine baths, irrigations, crenotherapy, radon treatments, sulphide baths, iodine–bromine baths, and peat-based therapies shows beneficial effects in the treatment of chronic pelvic pain, vulvodynia, and endometriosis, as well as in supporting the treatment of infertility. Peloidotherapy plays a particular role in regulating endocrine functions, improving tissue perfusion, and modulating the inflammatory response.

Pelvic floor muscle training, manual therapy, electrostimulation (including PTNS), magnetostimulation, shortwave diathermy, and monopolar radiofrequency contribute to reducing symptoms of urinary incontinence, dyspareunia, chronic pelvic pain, and sexual dysfunctions. A multimodal approach increases therapeutic efficacy through the synergistic action of different techniques.

Dietary modification plays a key role in the prevention and adjunctive treatment of diseases such as uterine fibroids, endometriosis, and polycystic ovary syndrome (PCOS). A diet rich in fruit (especially citrus), vegetables, vitamin D, and omega-3 fatty acids (in selected indications) may favourably influence hormonal and metabolic parameters. Limiting the consumption of red meat and adopting a Mediterranean diet are associated with improvements in sexual function and fertility parameters.

## Data Availability

No new data were created.

## Abbreviations

The following abbreviations are used in this manuscript:

25OHD	25-hydroxyvitamin D
ALA	alpha-lipoic acids
CPP	chronic pelvic pain
CRMRF	capacitive–resistive monopolar radiofrequency
CRP	c-reactive protein
DHA	docosahexaenoic acid
ExMI	extracorporeal magnetic innervation
FAI	free androgen index
FSFI	female sexual function index
GC	granulosa cells
GnRH	gonadotropin-releasing hormone
hs-CRP	high-sensitivity c-reactive protein
IL-1	interleukin-1
LH	luteinising hormone
NMES	electrical stimulation
OST	ozone sauna therapy
PCOS	polycystic ovary syndrome
PEMF	pulsed electric field therapy
PTNS	tibial nerve stimulation
SHGB	sex hormone-binding globulin
TAC	total antioxidant capacity
TENS	transcutaneous electrical nerve stimulation
TNF-α	tumour necrosis factor alpha
TT	total testosterone
